# Haploidentical hematopoietic cell transplant recipient presents with late‐onset Epstein Barr virus‐associated posttransplant lymphoproliferative disorder

**DOI:** 10.1002/jha2.707

**Published:** 2023-05-07

**Authors:** Adam Braun, Lawrence Liu, Monzr M. Al Malki, Pamela S Becker

**Affiliations:** ^1^ Hematology City of Hope Comprehensive Cancer Center Duarte California USA

**Keywords:** ebstein barr virus, haploidentical, hematopoietic stem cell transplant, leukemia, post transplant lymphoproliferative disorder, PTCy

## Abstract

Posttransplant lymphoproliferative disease (PTLD) is a potentially life‐threatening complication of hematopoietic cell transplantation. With improvements in Epstein‐Barr virus (EBV) monitoring and supportive care, PTLD incidence has decreased throughout the history of bone marrow transplantation. It is rare to develop PTLD after the first year following transplant, across all donor categories. In this case, we hope to elucidate details that may have predisposed to this unusual presentation. We present the case of a 55‐year‐old gentleman with acute myeloid leukemia who underwent a haploidentical transplant for consolidation and presented with fatigue, lethargy and presumed septic shock nearly 7 years after transplant.

## 1 INTRODUCTION

Posttransplant lymphoproliferative disease (PTLD) is a potentially life‐threatening complication of hematopoietic cell transplantation. With improvements in EBV monitoring and supportive care, PTLD incidence has decreased throughout the history of bone marrow transplantation. It is rare to develop PTLD after the first year following transplant, across all donor categories [1]. In this case, we hope to elucidate details that may have predisposed to this unusual presentation.

### 1.1 Case Presentation

We present the case of a 55‐year‐old gentleman with acute myeloid leukemia (AML) who underwent a haploidentical transplant for consolidation and presented with fatigue, lethargy and presumed septic shock nearly 7 years after transplant.

His initial AML presented with white blood count 3 k/uL with 62% circulating blasts, hemoglobin 10.4 g/dL, and platelet count of 60 k/uL. A bone marrow biopsy was completed. The aspirate was not read for unknown reason, but the core showed 90% cellularity, 80% blasts and necrotic areas. Immunohistochemistry showed blasts positive for CD68, CD163, and lysosome. Flow cytometry showed 16% myelomonocytic blasts expressing CD4, CD15, CD33, CD38, CD56, Human Leukocyte Antigen DR isotype (HLA‐DR), CD64, and negative for CD34 and CD114.  Cytogenetics showed 46 XY with trisomy 8 and deletion 13. Fluorescence in situ hybridization (FISH) testing showed 4–5 copies of 8q22 consistent with pentasomy 8 and additional chromosome 3 in 6%. Molecular studies showed NPM1 mutation present with variant allele frequency of 6%. FMS‐like tyrosine kinase‐3 internal tandem duplication (FLT3‐ITD), tyrosine kinase domain (TKD), and c‐Kit protooncogene (C‐KIT) mutations were not detected. CEBPA was wild type.

He underwent induction with idarubicin and cytarabine (7+3) and salvage therapy with fludarabine, idarubicin, granulocyte‐colony stimulating factor (G‐CSF) Fludarabine, Cytarabine, Idarubicin and G‐CSF (FLAG‐IDA) to achieve complete remission with incomplete count recovery (CRi). He had no recovery of counts after 4 months off therapy. Subsequently, he underwent haploidentical transplant with a conditioning regimen consisting of fludarabine, cyclophosphamide, and 1400 cGy total marrow and lymphoid irradiation, followed by posttransplant cyclophosphamide 50 mg/kg dosed daily on posttransplant days 3 and 4 prior to starting tacrolimus and mycophenolate mofetil. His course of transplant was complicated by *Clostridioides difficile* colitis, grade 2 hand‐foot syndrome, chemotherapy‐induced hemorrhagic cystitis without B.K. human polyomavirus (BK) or adenovirus isolated, cytomegalovirus (CMV) reactivation, grade 2 acute GVHD (GI only), disseminated *Varicella zoster* (VZV) complicated with severe postherpetic neuralgia, and deep venous thrombosis of the left lower extremity. Otherwise, he had 99.95% engraftment on peripheral blood with 100% engraftment by CD3 and CD15 donor cells by day 21. Lymphocyte subset flow cytometry on day 336 is included in Table 1. Notably, he had no evidence of EBV prior to transplant or in the following 2 years.

**TABLE 1:** Engraftment analysis at day 336 of T/B/NK lymphocyte subsets from peripheral blood.

Percent T‐cell (CD3+) count (56%–86%)	74
Absolute T‐cell (CD3+) count (723–2737 cells/uL)	3137
Percent T suppressor cell (CD3+CD8+) count (13–39%)	68
Absolute T suppressor cell (CD3+CD8+) count (220–1129 cells/uL)	2799
Percent T helper cell (CD3+CD4+) count (33%–58%)	6
Absolute T helper cell (CD3+CD4+) count (401–1612 cells/uL)	228
Helper/Suppressor ratio (0.7–3.6)	0.1
Percent NK cell (CD16+CD56+CD3‐) count (5%–26%)	4
Absolute NK cell (CD16+CD56+CD3‐) count (84–724 cells/uL)	178
Percent B‐cell (CD19+) count (5%–22%)	23
Absolute B‐cell (CD19+) count (80–616 cells/uL)	996

**FIGURE 1:**
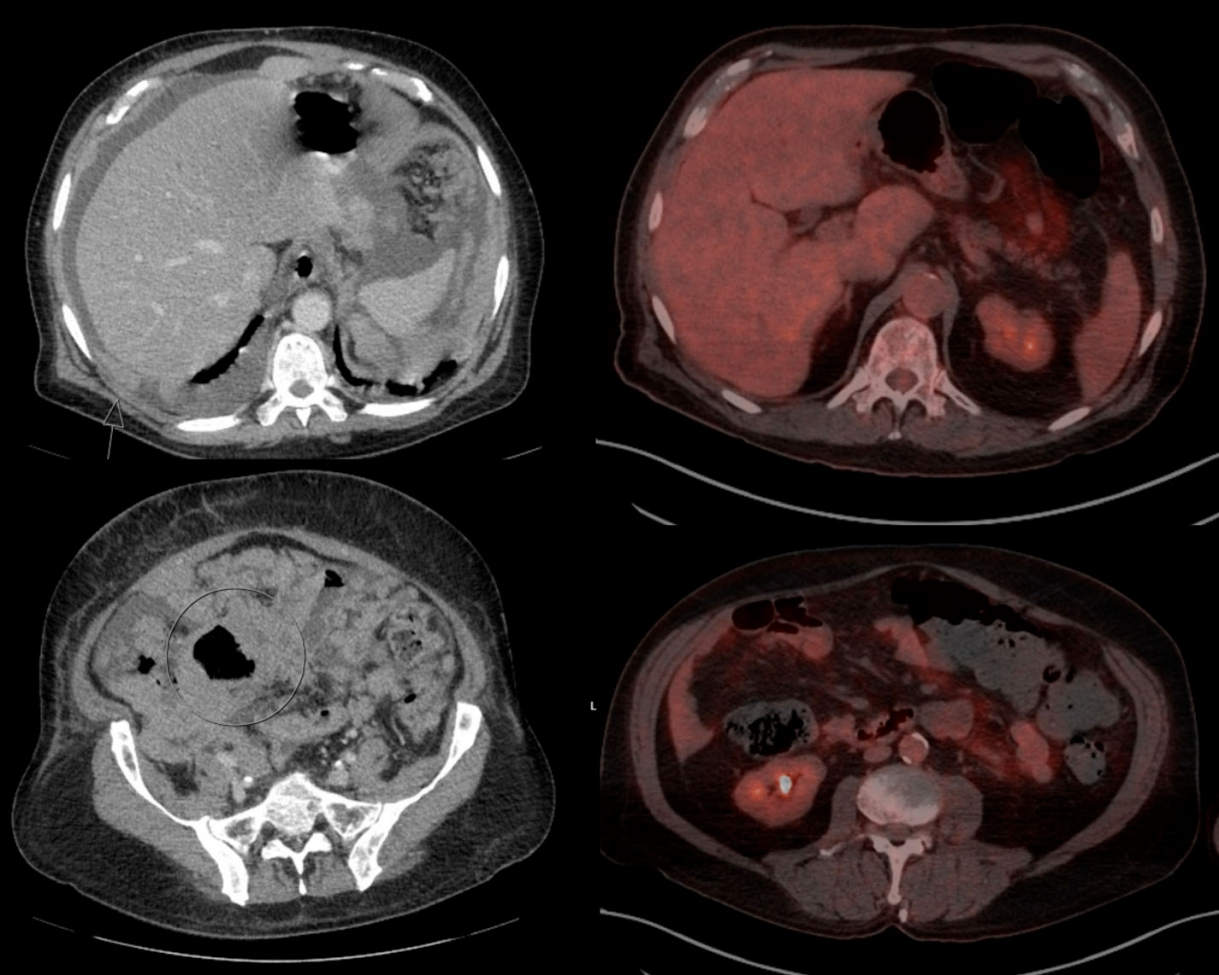
(Left) Initial CT chest, abdomen, and pelvis with contrast demonstrating nodular lymphadenopathy throughout the pleural and peritoneal cavity, including the diaphragm and circumferential distal colonic involvement. Measurable lymphadenopathy notable at the porta hepatis measuring approximately 40 × 29 mm and at the cardiophrenic interface measuring approximately 4.6 × 2.1 cm. (Right) Follow up positron emission tomography and computed tomography (PET/CT) demonstrating complete remission after 2 cycles of DA‐R‐EPOCH.

Six years and 10 months later, he presented with fatigue, lethargy, abdominal distention, and fever. Computerized tomography (CT) chest, abdomen, and pelvis with contrast revealed diffuse lymphadenopathy, as seen in Figure 1, with multiple sites of intestinal partial obstruction and splenic infiltration. After a few days, he developed acute dyspnea and hypotension and was intubated in the intensive care unit (ICU) for presumed septic shock. CT‐guided biopsy showed EBV‐positive Burkitt lymphoma with FISH positive for MYC:IGH fusion, MYC rearrangement, loss of BCL‐2 and BCL6, and loss of CEP 8. Bone marrow biopsy showed 50%–60% cellularity with no evidence of lymphoma or leukemia. Patient was treated urgently due to tumor lysis with hyperuricemia to 16.4 mg/dL, creatinine 2.81 mg/dL (baseline of 0.90 mg/dL) and lactate dehydrogenase of 5,054 U/L after high dose cytarabine x1 day for tumor burden reduction prior to the results of the biopsy. The following day, after confirmation of diagnosis, R‐EPOCH was started. Given the history of viral infections and new rash suggestive of VZV per dermatology consultant, high dose acyclovir 10 mg/kg three times per day (TID) was given for the first cycle. Therapy was complicated by bilateral deep vein thrombosis (DVT) lower extremities that resolved with enoxaparin. There was rapid improvement in his clinical course, and he was discharged on day 16 of the chemotherapy cycle. After 2 cycles, circulating EBV titers decreased from 2,098,745 on presentation copies to <250 copies, below the limit of detection. At the time of this report, he has completed three cycles of R‐EPOCH and prophylactic intrathecal chemotherapy with resolution of EBV quantitative Polymerase chain reaction (PCR) level and achieved CR by imaging.

## 2 DISCUSSION

Posttransplant lymphoproliferative disorder affects the immunocompromised hematopoietic cell and solid organ transplant population. This case was instructive for how late‐onset presentations of PTLD, although rare, must be recognized and treated promptly. A literature review was performed for other such cases of late‐onset PTLD and modern evidence‐based strategies to mitigate incidence of PTLD.

### 2.1 Epidemiology and risk factors

In the case of haploidentical transplant recipients with excessive immunosuppressive therapy as used in the Chinese experience, PTLD rarely presents after the first year [1]. There are many risk factors for the development of PTLD, including graft versus host disease grade 2 or greater, HLA disparity (unrelated donor or haploidentical donor), in vitro T cell–depleted antithymocyte globulin (ATG) or anti‐CD3 antibody in the conditioning regimen [2, 4, 5]. Each aforementioned strategy increases the likelihood of immunosuppression and opportunities for EBV infection. PTLD risk increases with the number of present risk factors in each case [2]. In this 1999 study of 18,014 patients who received any type of hematopoietic cell transplantation, only six patients had documented PTLD between 2.5 and 10 years posttransplant; none occurred beyond 10 years [2, 4]. Studies of patients who have undergone haploidentical hematopoietic cell transplantation (HCT) with posttransplantation cyclophosphamide (PTCy) prophylaxis for GvHD have repeatedly shown a dramatic decrease in incidence of PTLD, with no cases identified within the first 12 months [3, 4, 5]. This improves upon previously reported incidence of PTLD of between 4% and 8% in patients receiving unrelated donor or haploidentical HCT and ATG prophylaxis with or without selective T cell depletion [4, 5, 6].

### 2.2 Establishing the diagnosis

EBV is implicated in the development of PTLD, and non‐EBV‐associated posttransplant lymphoproliferative disorders in transplant are not considered PTLD per european conference on infections in leukemia (ECIL) guidelines [7, 8]. Early posttransplant, routine serologic studies of EBV DNA are recommended and expected to be noticed within the first 4 months, if present. EBV DNA testing does not confirm a diagnosis of PTLD. Common symptoms of fevers, tonsillitis, diarrhea, abdominal pain, lymphadenopathy, and hepatosplenomegaly are suggestive [9]. Staging can be completed in accordance with Ann Arbor and/or Lugano classification [8]. As continued improvements in immunosuppressive management and widespread implementation of PTCy, we expect ongoing progress in the reduction of incidence of PTLD.

### 2.3 Patient‐specific discussion

In our patient, who had been treated with posttransplantation cyclophosphamide, the late presentation of PTLD is unusual. He presented with typical symptoms, like lethargy, fatigue, and systemic inflammation [2, 3, 7]. The clear risk factors in his case include his severe immunodeficiency in the posttransplant period, as evidenced by CMV and VZV viremia, helper T cell lymphopenia[6], along with grade II acute GvHD, although chronic GvHD and use of cyclosporine conferred the highest risk for late‐onset PTLD in prior studies [5]. The PTLD in this case was suspected within 48 h of admission and empirically treated 5 days after admission. Clinical, serologic and radiologic testing all showed improvement and even complete remission within two cycles of treatment. He appears to be responding well to conventional dose‐adjusted R‐EPOCH therapy.

## 3 CONCLUSION

Recognizing signs and symptoms of PTLD is critical to early diagnosis and management, especially with late presentations. Current strategies like PTCy and weaning immunosuppression have reduced the incidence of PTLD. Future studies about the epidemiology and management of delayed or late‐onset PTLD will be needed to guide treatment in these scenarios, although these studies will likely be limited by the rare presentation of such patients.

## AUTHOR CONTRIBUTIONS

*Writing – original draft, review and editing*: Adam Braun. *Writing – review and editing*: Lawrence Liu. *Writing – review and editing, and supervision*: Monzr Al Malki. *Writing – review and editing, and supervision*: Pamela Becker.

## CONFLICT OF INTEREST STATEMENT

All the authors declare that they have no relevant conflict of interest to disclose.

## FUNDING INFORMATION

The authors received no specific funding for this work.

## ETHICS STATEMENT

This article does not contain any studies with human participants or animals performed by any of the authors. Informed consent was obtained from all individual participants included in the study. No material was reproduced from other sources. Case discussed was not part of a clinical trial

## Data Availability

Data cannot be shared openly to protect participant privacy. As this is a case report, data are stored on the City of Hope electronic medical record and would only be available for review by those directly involved in patient care or on a consented future study.

## References

[1] Xu L‐P , Zhang C‐Li , Mo X‐D , Zhang X‐H , Chen H , Han W , et al. “Epstein‐Barr virus–related post‐transplantation lymphoproliferative disorder after unmanipulated human leukocyte antigen haploidentical hematopoietic stem cell transplantation: incidence, risk factors, treatment, and clinical outcomes.” Biology of Blood and Marrow Transplantation. 2015;21(12):2185–91, ISSN 1083–8791. 10.1016/j.bbmt.2015.07.035 26253005

[2] Curtis RE , Travis LB , Rowlings PA , Socié G , Kingma DW , Banks PM , et al. “Risk of lymphoproliferative disorders after bone marrow transplantation: a multi‐institutional study” Blood. 1999;94:2208–16 10498590

[3] Liu Li , Zhang X , Feng S . “Epstein‐Barr virus‐related post‐transplantation lymphoproliferative disorders after allogeneic hematopoietic stem cell transplantation.” Biology of Blood and Marrow Transplantation. 2018;24(7):1341–9, ISSN 1083–8791. 29530767 10.1016/j.bbmt.2018.02.026

[4] Kanakry JA , Kasamon YL , Bolaños‐Meade J , Borrello IM , Brodsky RA , Fuchs EJ , et al. “Absence of post‐transplantation lymphoproliferative disorder after allogeneic blood or marrow transplantation using post‐transplantation cyclophosphamide as graft‐versus‐host disease prophylaxis.” Biol Blood Marrow Transplant. 2013; 19:1514–7 23871780 10.1016/j.bbmt.2013.07.013 PMC4051232

[5] Landgren O , Gilbert ES , Rizzo JD , Borrello IM , Brodsky RA , Fuchs EJ , et al. “Risk factors for lymphoproliferative disorders after allogeneic hematopoietic cell transplantation” Blood. 2009;113:4992–001 19264919 10.1182/blood-2008-09-178046 PMC2686146

[6] Long HM , Meckiff BJ , Taylor GS . The T‐cell response to Epstein‐Barr virus‐new tricks from an old dog. Front Immunol. 2019;10:2193. 10.3389/fimmu.2019.02193. PMID: 31620125; PMCID: PMC6759930. 31620125 PMC6759930

[7] Compagno F , Basso S , Panigari A , Bagnarino J , Stoppini L , Maiello A , et al. Management of PTLD after hematopoietic stem cell transplantation: immunological perspectives. Front Immunol. 2020;11:567020. 10.3389/fimmu.2020.567020. PMID: 33042147; PMCID: PMC7526064. 33042147 PMC7526064

[8] Styczynski J , van der Velden W , Fox CP , Engelhard D , de la Camara R , Cordonnier C , et al. Sixth European Conference on Infections in Leukemia, a joint venture of the Infectious Diseases Working Party of the European Society of Blood and Marrow Transplantation (EBMT‐IDWP), the Infectious Diseases Group of the European Organization for Research and Treatment of Cancer (EORTC‐IDG), the International Immunocompromised Host Society (ICHS) and the European Leukemia Net (ELN). Management of Epstein‐Barr virus infections and post‐transplant lymphoproliferative disorders in patients after allogeneic hematopoietic stem cell transplantation: Sixth European Conference on Infections in Leukemia (ECIL‐6) guidelines. Haematologica. 2016;101(7):803–11. 10.3324/haematol.2016.144428. PMID: 27365460; PMCID: PMC5004459. 27365460 PMC5004459

[9] Dierickx D , Habermann TM . Post‐transplantation lymphoproliferative disorders in adults. N Engl J Med. 2018;378(6):549–62. 10.1056/NEJMra1702693 29414277

